# Do SGLT2 Inhibitors Improve Cardio-Renal Outcomes in Patients With Type II Diabetes Mellitus: A Systematic Review

**DOI:** 10.7759/cureus.17668

**Published:** 2021-09-02

**Authors:** Sahithi Reddy Kalluri, Tinaz H Bhutta, Hanan Hannoodee, Mahmoud Al Khalili, Nyein Wint Yee Theik, Oluwatimilehin E Raji, Priya Shenwai, Rutul Shah, Safeera Khan

**Affiliations:** 1 Internal Medicine, California Institute of Behavioral Neurosciences & Psychology, Fairfield, USA; 2 Internal Medicine, California Institute of Behavioral Neurosciences & Psychology, Farfield, USA

**Keywords:** cardiovascular disease, urinary tract infection, luseogliflozin, type 2 diabetes, sodium-glucose cotransporter-2 (sglt2) inhibitors, kidney disease, empagliflozin, dapagliflozin, canagliflozin

## Abstract

Diabetes mellitus (DM) is associated with dreadful changes in the cardiovascular and renal systems, causing increased morbidity and mortality. Sodium-glucose cotransport-2 (SGLT2) inhibitors belong to the oral hypoglycemic group of drugs believed to reduce these events by various mechanisms in DM. We performed a systematic review to determine the effectiveness of SGLT2 inhibitors in reducing cardiovascular and renal complications and address safety concerns in participants with type 2 diabetes mellitus (T2DM).

We explored PubMed, PubMed Central, Medical Literature Analysis and Retrieval System Online (MEDLINE), Cochrane library, and ResearchGate for randomized controlled trials and observational studies done on the advantages of SGLT2 inhibitors in the prevention or reduction of worsening cardiovascular and renal changes in T2DM. Studies were screened for the quality assessment using the Cochrane risk-of-bias assessment tool and Newcastle-Ottawa scale. We screened 5615 articles, out of which 22 articles with 7,02,977 diabetes mellitus patients treated with SGLT2 inhibitors were used for the systematic review after meticulously filtering articles based on inclusion and exclusion criteria. The trials included one of the following drugs - empagliflozin, dapagliflozin, canagliflozin, and luseogliflozin. SGLT2 inhibitors significantly reduced the risk of heart failure (HF), frequency of hospitalizations due to HF, all-cause mortality, cardiovascular mortality, and nonfatal myocardial infarction. Renal outcomes showed a significant lowering of risk of acute kidney failure, progression of chronic kidney disease, renal mortality, and improvement in urinary albumin creatinine ratio. We noticed an initial worsening of the estimated glomerular filtration rate followed by stabilizing and reaching the baseline on long-term treatment, especially in end-stage renal failure patients.

The review showed that SGLT2 inhibitors have adverse reactions similar to that of a placebo, with a slight increase in treatable genital mycotic and urinary tract infections but no evidence of diabetic ketoacidosis, fractures, and amputations. According to the available data, SGLT2 inhibitors can significantly prevent or reduce cardiovascular diseases and kidney abnormalities in patients with type 2 diabetes mellitus with tolerable safety outcomes.

## Introduction and background

Centre for Disease Control and Prevention (CDC) data states that as of 2018, diabetes mellitus (DM) in the USA is prevalent in approximately 10.5% of the population, with serious microvascular and macrovascular complications [1]. Significant microvascular changes cause chronic kidney disease (CKD) with a prevalence of 43.2% in type 2 diabetes mellitus (T2DM) cases [2]. Cardiovascular disease (CVD) is also a major complication affecting 32.2% of DM type II patients and the cause of death in 50.3% of patients [3].

Sodium-glucose cotransporter-2 (SGLT2) inhibitors, including empagliflozin, dapagliflozin, and canagliflozin, are approved for glucose lowering in patients with T2DM along with reducing reno-vascular complications and enhancing weight loss. The mechanism of action of SGLT2 inhibitors is to promote glucose excretion in urine by blocking glucose and sodium reuptake in the early proximal renal tubule and thereby reduce blood glucose levels [4]. The other beneficial effect of SGLT2 inhibitors is their promotion of sodium excretion in urine that causes osmotic diuresis and reduced blood pressure [4,5]. These drugs reduce endogenous glycerol-derived hepatic gluconeogenesis from visceral adipose tissue causing weight loss [6]. One of the renal protective mechanisms of SGLT2 inhibitors is in albuminuria where it reduces intra-glomerular pressure and prevents subsequent tubular cell injury [7]. These pleiotropic effects have reduced cardiovascular events and preserved kidney functions. According to American Diabetes Association (ADA), these drugs are currently recommended as supplement treatment in T2DM patients with high risk or established CVD, established CKD, or heart failure (HF) independent of hemoglobin A1C (HbA1C) values of the patients [8].

There is no sufficient evidence showing glucose-lowering medications reduce the risk of cardiovascular disorders or mortality in T2DM patients. A moderate cardiovascular benefit may be observed after long-term treatment and follow-up as noticed in a few studies [9]. Besides, there may be adverse cardiovascular outcomes with strict glycemic control or due to the use of SGLT2 inhibitors [10]. 

SGLT2 inhibitors affect the kidney; they might pose a threat to patients with renal disorders. The function of the drug depends on the renal function/filtration capacity of the kidney; as kidney function declines, the efficacy of the drug declined [11]. The risk-benefit ratio should be assessed before starting treatment with these drugs. SGLT2 inhibitors are not currently advised in chronic kidney disease patients with an estimated glomerular filtration rate (eGFR) of <45mL/min/1.73m2 for empagliflozin and canagliflozin, and <60mL/min/1.73m2 for dapagliflozin [12,13,14]. This raises queries about the effects on efficacy to improve cardiovascular, renal, and safety outcomes in people with reduced eGFR.

Hence, we started a systematic review of randomized controlled trials and observational trials to understand better the efficiency of SGLT2 inhibitors in providing protection from cardiovascular disorders and preventing renal adverse outcomes in individuals with T2DM.

## Review

Methods

**Figure 1:**
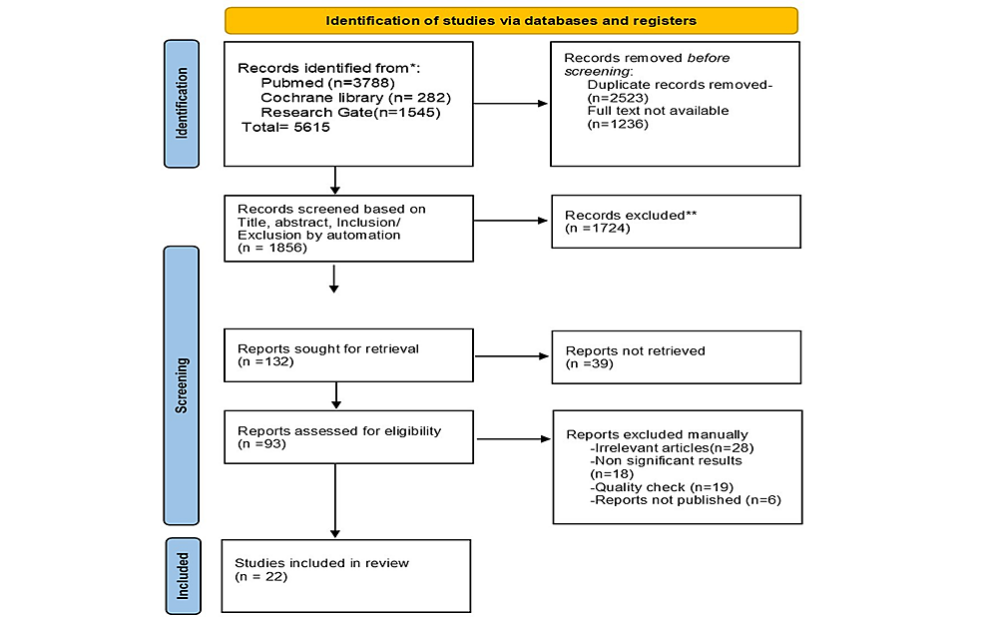
Preferred Reporting Items for Systematic Reviews and Meta-Analyses (PRISMA) flow diagram

We conducted a systematic review as per Preferred Reporting Items for Systematic Reviews and Meta-Analyses (PRISMA) guidelines [15]. Figure 1 depicts the search process in the form of a PRISMA flow diagram.

Data Source and Strategy

We systematically searched for relevant articles indexed at PubMed, PubMed Central, MEDLINE, Cochrane library, and ResearchGate. We explored the databases using keywords and the medical subject headings (MeSH) strategy to maximize the number of articles.

We used the following MeSH strategy to seach for relevant papers: ("Sodium-Glucose Transporter 2 Inhibitors/administration and dosage"[Mesh] OR "Sodium-Glucose Transporter 2 Inhibitors/adverse effects"[MeSH] OR "Sodium-Glucose Transporter 2 Inhibitors/therapeutic use"[MeSH] OR "Sodium-Glucose Transporter 2 Inhibitors/toxicity"[MeSH] ) OR ( "Canagliflozin/administration and dosage"[Majr] OR "Canagliflozin/adverse effects"[Majr] OR "Canagliflozin/therapeutic use"[Majr] OR "Canagliflozin/toxicity"[Majr] ) OR ("Sodium-Glucose Transporter 2 Inhibitors" [Pharmacological Action]) AND ("Diabetes Mellitus, Type 2/drug therapy"[Majr] OR "Diabetes Mellitus, Type 2/therapy"[Majr] ) AND ("Heart Failure/drug therapy"[Majr] OR "Heart Failure/prevention and control"[Majr] OR "Heart Failure/therapy"[Majr] ) AND ("Renal Insufficiency, Chronic/drug therapy"[Majr] OR "Renal Insufficiency, Chronic/prevention and control"[Majr] OR "Renal Insufficiency, Chronic/therapy"[Majr]).

Inclusion and Exclusion Criteria

We included peer-reviewed randomized controlled trials (RCTs) and observational studies relevant to the research question published in the English Language in the past 10 years that focus on the adult and geriatric population (>18years).

We excluded articles published in other languages, grey literature, books, case series, case reports, unpublished literature, and the pediatric population.

Screening and Quality/Bias Assessment

We assessed 37 studies for quality using standardized quality assessment tools, and 33 articles qualified for the quality appraisal. The following tools were used:

1. Randomized controlled trials: Cochrane risk-of-bias assessment tool.

2. Observational studies: Newcastle-Ottawa scale

Results

Our initial search yielded 5615 articles. After the duplicates (n=2523) and those that weren't available with full text (n=1236) were removed, we were left with 1856 articles that were screened by title and inclusion/exclusion criteria by automation. Following this, 1724 non-relevant articles were excluded. Out of the remaining 132 articles, 39 articles could not be retrieved. Abstracts and full text of the 93 relevant articles that remained were thoroughly read, and 52 were excluded after inclusion criteria were applied. Lastly, after meticulous quality assessment, 19 more articles were excluded.

Our review article includes 22 articles with 7,02,977 diabetes mellitus patients from 18 randomized control trials, and four observational studies that included one of the following drugs - empagliflozin, dapagliflozin, canagliflozin, and luseogliflozin. SGLT2 inhibitors significantly reduced the risk of HF, frequency of hospitalizations due to HF, all-cause mortality, cardiovascular (CV) mortality, and nonfatal myocardial infarction. Renal outcomes showed a significant lowering of risk of acute kidney failure, progression of CKD, renal mortality, and improvement in urinary albumin creatinine ratio. Studies showed that adverse reactions in both SGLT2 inhibitors and placebo groups were identical, with a slight increase in treatable genital mycotic and urinary tract infections in the treatment group. There was no evidence of diabetic ketoacidosis, fractures, and amputations.

Discussion

SGLT2 inhibitors, when used in patients with diabetes mellitus, can reduce the risk for cardiac and renal abnormalities significantly but with a debatable safety profile.

Cardio-Protective Profile of SGLT2 Inhibitors

**Figure 2:**
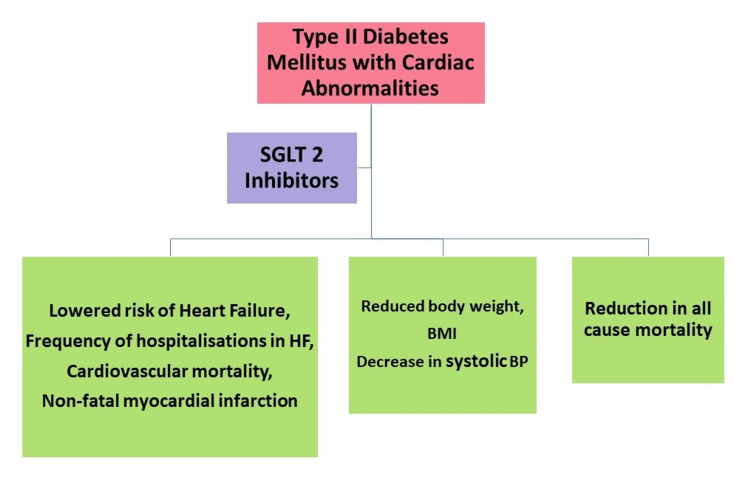
Describes the uses of SGLT2 inhibitors in type 2 diabetes mellitus patients with heart disorders SGLT2: Sodium-glucose cotransporter-2 inhibitors; HF: Heart failure, BMI: Body mass index; BP: Blood pressure

With cardiovascular disorders being an important cause of mortality in T2DM patients, the efficacy of the SGLT2 inhibitor to reduce this risk is assessed. An international study by Kosiborod et al. conducted in patients with T2DM from the Asia Pacific, the Middle East, and North America, comparing SGLT2 inhibitors and other glucose-lowering drugs, showed a significant reduction in hospitalization due to HF, the incidence of myocardial infarction (MI), and all-cause mortality [16]. A randomized control trial by Zinman et al. on 7020 patients found that using the empagliflozin drug lowered the risk of death from cardiovascular complications in comparison to the placebo [17]. Fitchett et al. stated that empagliflozin could reduce the risk of recurrence and mortality of MI/stroke in patients with type 2 diabetes mellitus but the results were insignificant [18]. An overview of the cardiac-related advantages of SGLT2 inhibitors in T2DM patients is given in Figure 2.

Post hoc analysis of the empagliflozin cardiovascular outcome event trial in type 2 diabetes mellitus patients-removing excess glucose (EMPA-REG) done in 2020 by Kaku et al., studied the effect of empagliflozin vs. placebo in reduction of seven cardiovascular (CV) risk factors. Participants were classified into three subgroups based on their risk factor score of zero to three, four to five, or six to seven of the following seven goals for CV risk factor control at baseline: (1) HbA1C <7.5%; (2) low density lipoprotein cholesterol <100mg/dL or statin use; (3) systolic blood pressure (SBP) <140mmHg and diastolic blood pressure (DBP) <90mmHg; (4) use of angiotensin-converting enzyme (ACE) inhibitor or angiotensin II receptor blocker; (5) normoalbuminuria; (6) aspirin use; (7) non-smoking status. Results showed that 56% had achieved four or five goals at the time of the study, 31% had met six, and only 6.7% had achieved all seven goals [19]. Kaku et al. discussed the effect of empagliflozin in 1517 Asian patients with CV risk factors and T2DM; the study showed that three-point major adverse cardiac events (MACE), four-point MACE, hospitalization for HF, the composite of HF hospitalization or CV death were significantly decreased and were consistent with the other studies [20].

A recent RCT by Phrommintikul et al. comparing dapagliflozin and vildagliptin showed a significant reduction in body weight and body mass index (BMI) in the dapagliflozin group [21]. In contrast, a significant increase in weight was observed in the vildagliptin. Systolic blood pressure was significantly decreased in the dapagliflozin group but did not change in the vildagliptin group. There was no significant change in diastolic BP, heart rate, eGFR, N-terminal pro-brain natriuretic peptide (NT-proBNP) in either group. The mean hemoglobin was significantly increased in the dapagliflozin group. In contrast, no change was seen in the vildagliptin group. High-sensitivity Troponin T (Hs-TnT) significantly decreased in the dapagliflozin group, though a non-significant increase in Hs‐TnT was noticed in the other group [21].

A large CANVAS study program by Rådholm et al. was conducted on 10,142 patients with T2DM. In this study, compared to the placebo, canagliflozin reduced the risk of cardiovascular death or hospitalization due to heart failure significantly in patients with a previous history of HF, wheras in patients with no history of HF at baseline, the results showed p-value>0.05 after a mean follow-up time of 188.2 weeks [22]. Table 1 shows the synopsis of 10 articles depicting the cardiovascular effects of SGLT2 inhibitors.

**Table 1:** Summary of the articles which demonstrate the efficacy of SGLT2 inhibitors in T2DM patients to improve cardiac abnormalities. T2DM: Type 2 diabetes mellitus; CV: Cardiovascular; SGLT2i: Sodium-glucose cotransporter-2 inhibitors; HHF: Hospitalization due to heart failure; MI: Myocardial infarction; MACE: Major adverse cardiac effects; CAD: Coronary artery disease; CKD: Chronic kidney disease; CVD: Cardiovascular disease; AF: Atrial fibrillation

Publication	Year	Drug	Patients	Type of Study	Purpose	Results/Conclusion
Kosiborod et al. [16]	2018	All	235,064	RCT	Outlined CV outcomes in patients using SGLT2i and OGLDs across six countries	SGLT2i group showed a reduced risk of death, HHF, MI vs. OGLDs
Zinman et al. [17]	2017	Empagliflozin	7020	RCT	Effects on cardiovascular morbidity and mortality in T2DM	Lower rate of death from cardiovascular causes, nonfatal MI, or nonfatal stroke
Fitchett et al [18]	2019	Empagliflozin	10,773	RCT	Examined empagliflozin effects on 3-point MACE, CVD and all-cause death, and HHF across various cardiovascular risk groups	Reductions in CV outcomes and mortality were similar across the spectrum of cardiovascular risk
Inzucchi et al. [19]	2020	Empagliflozin	7020	RCT	To identify the association of CV risk factor reduction with CV benefits of empagliflozin	The cardioprotective effect was consistent regardless of multiple baseline risk factor control
Kaku et al. [20]	2017	Empagliflozin	7,020	RCT	Effects of empagliflozin in patients of Asian race	MACE, all-cause mortality, and HF outcomes in Asian patients were congruous with the worldwide population
Phrommintikul et al. [21]	2019	Dapagliflozin	49	RCT	Investigate cardiometabolic effects of dapagliflozin and vildagliptin in T2DM patients with CAD	More benefits in cardiovascular outcomes were observed in dapagliflozin compared to vildagliptin
Radholm et al. [22]	2018	Canagliflozin	10,142	RCT	Identify the advantages of canagliflozin on HF and cardiovascular death overall, in those with and without a history of HF	The benefit of reduced mortality and HHF may be higher in patients with a past history of HF
Mahaffey et al. [23]	2019	Canagliflozin	4401	RCT	Effect of canagliflozin to reduce adverse CV outcomes in T2DM and CKD patients with or without previous CVD	Canagliflozin significantly decreased MACE and kidney failure, including the participants with no previous CVD
Bohm et al. [24]	2020	Empagliflozin	7,020	Observational	Explore CV and renal outcomes in patients with vs. without AF at baseline	Empagliflozin lowered HF-related and renal events, irrespective of AF status
Chen HY et al. [25]	2020	All	399,810	Observational	Identify the link between SGLT2 inhibitors and the risk of arrhythmias	SGLT2 inhibitors were associated with a reduced risk of all-cause mortality and new-onset arrhythmias

Data is available on the efficiency of SGLT2 inhibitors in improving cardiac parameters in patients diagnosed with cardiac abnormalities. Still, Mahaffey et al. discussed the use of SGLT2 inhibitors for primary prevention of cardiac disorders vs. secondary preventions of complications in patients with existing cardiac abnormalities in T2DM patients [23]. The primary group included 2181 patients, nearly 50% with no history of documented cardiovascular disease, and 2220 participants who had some previous cardiac diseases were included in the secondary prevention group. Surprisingly, canagliflozin reduced major cardiovascular events in both groups. These results were consistent in those with risk of cardiovascular death, nonfatal myocardial infarction, nonfatal stroke, or hospitalization for heart failure in both the primary and secondary prevention groups [23].

A retrospective analysis by Böhm et al. was done on the effect of empagliflozin on reducing cardiac adverse events in patients with T2DM with and without atrial fibrillation (AF). Increased cardiac abnormalities were noticed in the AF group at baseline, but empagliflozin consistently reduced the cardiac events in both groups significantly by reducing the plasma volume, load reduction on ventricles, and by enhancing cardiac energy. The event rates of nephropathy were similar in both groups, but the drug reduced incidence and progression of nephropathy in patients with AF vs. without AF by increasing sodium and fluid excretion causing a reduction in volume overload on kidneys [24]. Another large longitudinal study by Chen et al. on 399,810 patients with T2DM demonstrated that SGLT2 inhibitors were linked to significantly reduce risk of all-cause mortality and new-onset arrhythmias compared to participants that were not on any SGLT2 inhibitors [25].

Renal-Protective Profile of SGLT-2 Inhibitors

**Figure 3:**
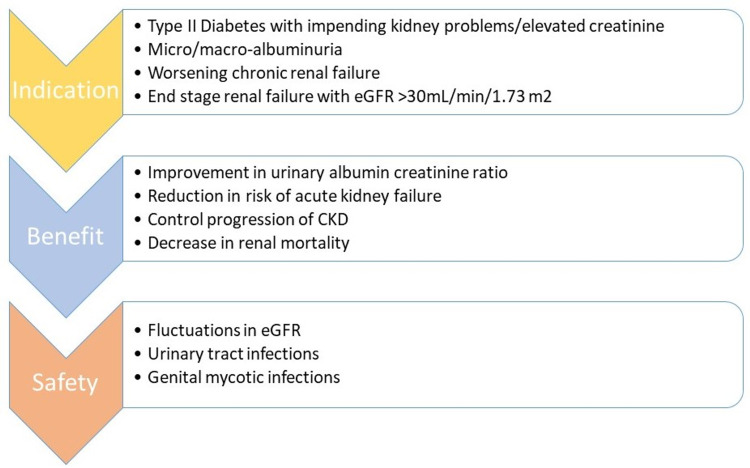
Emphasizes the indication, advantage, and adverse effects of SGLT2 inhibitors when used to improve renal function. CKD: Chronic kidney disease; eGFR: Estimated glomerular filtration rate

Reduction in urinary albumin creatinine ratio (UACR) is believed to be an important mechanism of action of SGLT2 inhibitors in improving renal outcomes. Oshima et al. stated that canagliflozin at the end of 26 weeks reduced geometric mean UACR by 31%, and also reduced the risk of progression in UACR from non-nephrotic to nephrotic-range albuminuria and regression in UACR stage from macroalbuminuria to micro or normoalbuminuria compared with placebo. These early albumin changes independently improve renal and cardiac disorders when used for the long term in participants with T2DM with CKD [26]. Figure 3 gives a brief outline of the usage of SGLT2 inhibitors to improve renal function.

Heerspink et al., in a trial on 4304 CKD patients, examined the efficacy of dapagliflozin, of which 32.5% did not have type 2 diabetes. Results showed a significant reduction in risk of constant downslope in the eGFR by at least 50%, end-stage kidney disease, or mortality from renal or cardiovascular causes with dapagliflozin compared to placebo, with results being similar in patients with or without T2DM [27]. Canagliflozin and renal events in diabetes with established nephropathy clinical evaluation (CREDENCE) trial is another large study conducted in 34 countries, which stated that the rate of the end-stage kidney disease progression, doubling of the serum creatinine level, or renal or cardiovascular death was significantly lower in the canagliflozin group than in the placebo group, with 30% lower relative risk with p-value=0.00001 [28]. A Japanese study on the effects of canagliflozin in patients with T2DM and HF showed improvements in renal function after 12 months after the initiation of treatment. This trial by Sezai et al. concluded that SGLT2 inhibitors could be used as first-line treatment in heart failure for T2DM patients with preserved ejection fraction [29].

Although studies showed improvement in renal function, long-term trials with frequent follow-up showed fluctuating eGFR results. Wanner et al. noticed significant relative risk reduction in incidence or worsening nephropathy, macroalbuminuria, doubling of creatinine in patients treated with empagliflozin vs. placebo groups. But no difference in the rate of incident albuminuria in both the groups. They observed a significant short-term reduction in eGFR in empagliflozin groups, compared with a small increase in the placebo group from baseline to week four (period one). After continued administration (period two), stabilization of eGFR in the SGLT2 group and progressive decline in placebo. Drugs were withdrawn during the last week of treatment to follow-up (period three), a significant increase in eGFR with both doses of empagliflozin. However, a small decrease in the placebo group was identified [30]. Similar findings were also discussed by a randomised prospective Japanese study on the reno-protective function of canagliflozin in T2DM with CKD patients, i.e., reduction in eGFR from baseline for four weeks but later improved to reach the baseline values by 26 weeks in canagliflozin group. Significant reduction in urinary albumin-creatinine ratio, urinary liver-type free acid-binding protein, N-acetyl-β-d-glucosaminidase, and β2-microglobulin levels was also observed in the treatment group vs. control group [31]. Eight studies that looked into the kidney protective action of SGLT2 inhibitors in T2DM patients are summarized in Table 2.

**Table 2:** Outlines the articles describing the function of SGLT2 inhibitors to improve renal outcomes. T2DM: Type 2 diabetes mellitus; CKD: Chronic kidney disease; SGLT2i: Sodium-glucose cotransporter-2 inhibitors; DPP-4i: Dipeptidyl peptidase 4 inhibitors

Publication	Year	Drug	Study Population	Type of Study	Purpose	Results/Conclusion
Oshima et al. [26]	2019	Canagliflozin	4401	RCT	To Identify the association between early changes in albuminuria and cardiorenal abnormalities	Early reductions in albuminuria were independently associated with long-term benefits in kidney and cardiovascular outcomes
Heerspink et al. [27]	2020	Dapagliflozin	4304	RCT	Effect of the dapagliflozin on renal and cardiovascular events in CKD patients with and without diabetes	Improvement in renal function and reduced mortality among patients with CKD, irrespective of DM status
Perkovic et al. [28]	2019	Canagliflozin	4401	RCT	To assess the renal outcomes in T2DM and CKD on long term use of SGLT2 inhibitors	Lower risk of kidney failure and cardiovascular events in the canagliflozin group
Sezai et al. [29]	2019	Canagliflozin	35	Clinical Trial	To demonstrate the cardioprotective function in T2DM and chronic heart patients	Canagliflozin improved oxidative stress, diastolic function, endothelial function, displayed cardiac and renal protective effects
Wanner et al. [30]	2016	Empagliflozin	7020	RCT	To determine the long-term renal effects of empagliflozin	In T2DM with high cardiovascular risk, empagliflozin showed delayed progression of CKD and low rates of renal events
Takashima et al. [31]	2018	Canagliflozin	40	RCT	To evaluate the renoprotective effects, albuminuria- lowering effects of canagliflozin, in Japanese in T2DM with CKD	Canagliflozin was associated with a slower progression of kidney disease. Reduction in albuminuria and tubulointerstitial markers.
Ito et al. [32]	2021	Luseogliflozin	238	Observational	The safety and efficacy of luseogliflozin in Japanese patients	Preserves the renal function in the medium term in patients with T2DM and renal impairment
Pasternak et al. [33]	2020	All	29,887	Observational	To assess the association between SGLT2i and DPP-4i to lower the risk of serious renal events	SGLT2 inhibitors were associated with a significantly reduced risk of serious renal events

In a retrospective study by Ito et al., patients were divided into three groups based on eGFR values to identify any variations between the groups, with a follow-up of 12 months before and after administration of luseogliflozin. Results showed preserved eGFR in patients with low eGFR group after administration. Still, they gradually decreased before the administration in the present study. In contrast, there was a slight decrease in eGFR in normal or high eGFR groups after the initiation of SGLT2 inhibitor treatment [32].

A large cohort study on 59,772 patients was done by Pasternak et al. to study and compare SGLT2 inhibitors. Dipeptidyl peptidase 4 (DPP-4) inhibitors showed the incidence of renal events was significantly lower in the SGLT2 group, which was more during the initial two years of follow-up. SGLT2 inhibitors were also associated with a significantly low risk of renal replacement therapy and hospital admission from renal events vs. DPP-4 inhibitors [33].

Safety Profile of SGLT-2 Inhibitors

SGLT2 inhibitors are well tolerated with a lower number but with serious adverse reactions. A 52-week double-blinded randomized control study by Damman et al. showed that mild to moderate intensity recurrent genital infections, single episodes of lower urinary tract infections (UTI) that responded to standard treatment were seen in greater proportions in the dapagliflozin vs. glipizide group. No events of pyelonephritis, diabetic ketoacidosis, and elevated liver enzymes were noted in the SGLT2 inhibitors group [34]. Another pilot study on empagliflozin also showed no difference in cases of UTI compared to placebo [35]. Upon further review, four studies were identified that discussed the adverse reactions of SGLT2 inhibitors, and these are summarized in Table 3.

**Table 3:** Synopsis of articles describing the adverse effects of SGLT2 inhibitors T2DM: Type 2 diabetes mellitus; SGLT2i: Sodium-glucose cotransporter-2 inhibitors; HF: Heart failure; CKD 3A: Chronic kidney disease stage 3A; UTI: Urinary tract infection; DKA: Diabetic ketoacidosis; eGFR: Estimated glomerular filtration rate

Publication	Year	Drug	Population	Study type	Purpose	Results/ conclusion
Nauck et al. [34]	2011	Dapagliflozin	814	RCT	Comparison of the efficacy, safety, and tolerability of dapagliflozin vs. sulfonylurea glipizide in T2DM	Dapagliflozin reduced weight and produced less hypoglycemia than glipizide in T2DM with similar efficacy
Damman et al. [35]	2020	Empagliflozin	80	RCT	Safety and clinical efficacy of SGLT2 inhibitors in patients with acute decompensated HF	Showed no difference in cases of UTI compared to placebo
Fioretto et al. [36]	2018	Dapagliflozin	321	clinical Trial	To assess the efficacy and safety of dapagliflozin 10mg vs. placebo in patients with T2DM and moderate renal impairment (CKD 3A)	No increase in adverse events, no bone fractures, amputations, or DKA were reported with dapagliflozin vs. placebo
Jardine et al. [37]	2020	Canagliflozin	4401	RCT	Described efficacy and safety of canagliflozin across various eGFR groups	No harmful adverse effects, amputations, and fractures. Results were consistent among eGFR subgroups.

**Figure 4:**
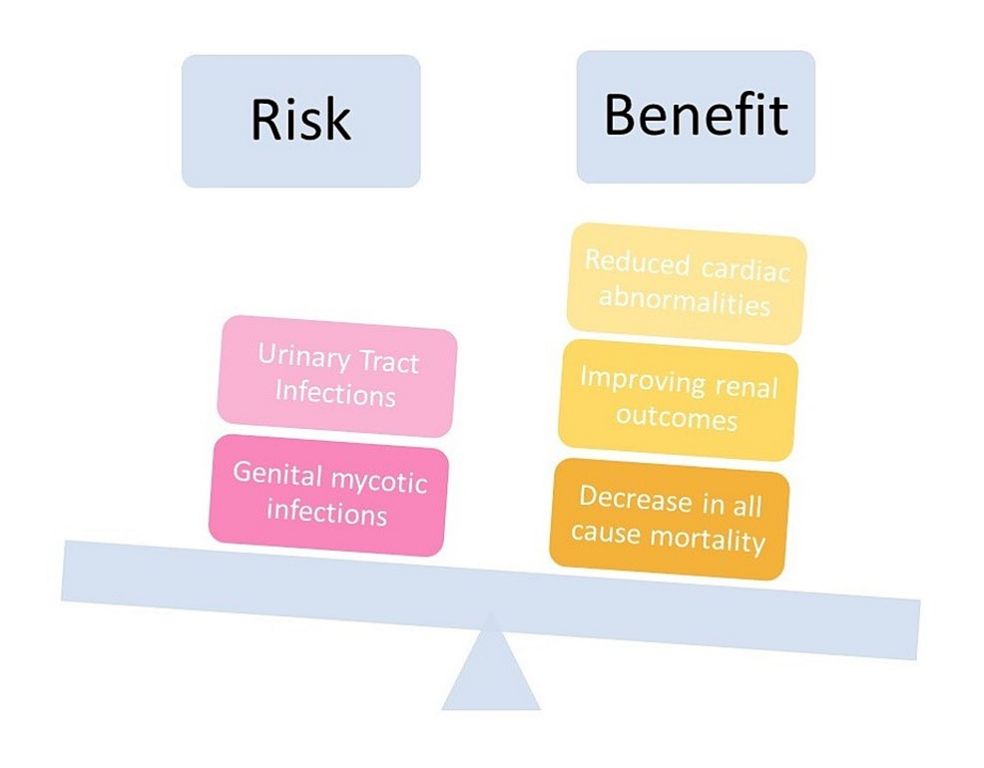
Denotes that the benefits of SGLT2 inhibitors out-weigh the risk of using the drug.

The study to evaluate the effect of dapagliflozin on blood glucose level and renal safety in patients with type 2 diabetes (DERIVE) reported no difference in percentages of genital infections, and UTI in dapagliflozin compared to the placebo group. However, the drug caused worsening of eGFR/renal failure for the initial four weeks and returned to baseline within 27 weeks. In contrast, hypoglycemia was rare in both the treatment and placebo groups. Occasionally hypotension, dehydration, hypovolemia was identified [36]. Jardine et al. stated that rates of fractures and amputations were similar in both canagliflozin and placebo groups irrespective of eGFR. Whereas the canagliflozin group showed significantly fewer cases of volume depletion and osmotic diuresis, variations were observed among different eGFR subgroups [37]. Figure 4 represents the cardiac and renal benefits that exceed the adverse effects of SGLT2 inhibitors in T2DM patients.

Limitations

Although our systematic review included multiple RCTs and observational studies which showed the significant role of SGLT2 inhibitors in reducing cardiac and renal outcomes with a good safety profile, there are certain limitations. One, the heterogeneity in the characteristics of the population, clinical setting of the study and follow-up duration of the studies included. Two, there are not enough studies regarding the cardio-renal protective role in the non-diabetic population. Three, a low cardiac risk population accounts for a significant proportion that was not included in these trials. Finally, data about the eGFR fluctuations and efficacy of SGLT2 inhibitors in CKD stage 3/4 should be addressed. The current large trials such as the study of heart and kidney protection with empagliflozin (EMPA-KIDNEY), Semaglutide ardiovascular outcomes trial in patients with type 2 diabetes (SOUL), and the study to see how semaglutide works compared to placebo in people with type 2 diabetes and chronic kidney disease (FLOW) may be necessary to provide better insight into these constraints.

## Conclusions

This systematic review is performed to consolidate the evidence of sodium-glucose cotransport-2 (SGLT2) inhibitors to reduce cardiovascular and renal complications and adverse effects in diabetes mellitus from 22 randomized control trials and observational studies. SGLT2 inhibitors significantly lowered all-cause and cardiovascular mortality, myocardial infarction, hospitalizations due to heart failure, the risk for new-onset arrhythmias, kidney failure, incidence or worsening nephropathy, macroalbuminuria, lower body weight, blood pressure with a slight increase in the risk of UTI, and genital infections in T2DM patients.

This data serves as an overview for physicians regarding various studies performed. These drugs can be used in T2DM patients with cardiovascular complications to reduce worsening in low to high-risk patients. But caution is required in CKD patients, with close monitoring of eGFR. More studies are required to provide evidence of the efficacy of SGLT2 inhibitors in non-diabetic patients with cardiac or renal diseases. Larger sample sizes and long-term RCTs, and observational studies are needed to know the prophylactic benefits of using SGLT2 inhibitors in de novo diabetes mellitus patients with no heart or kidney complications.
